# From women to women—hematuria during therapy for metastatic breast cancer, what to suspect and when to be alarmed; Bladder metastasis from breast cancer—our experience and a systematic literature review

**DOI:** 10.3389/fonc.2022.976947

**Published:** 2022-09-29

**Authors:** Rafaela Malinaric, Federica Balzarini, Giorgia Granelli, Arianna Ferrari, Giorgia Trani, Francesca Ambrosini, Guglielmo Mantica, Daniele Panarello, Aldo Franco De Rose, Carlo Terrone

**Affiliations:** ^1^ Department of Urology, L'Istituto di ricovero e cura a carattere scientifico (IRCCS) Ospedale Policlinico San Martino, Genova, Italy; ^2^ Department of Surgical and Diagnostic Integrated Sciences (DISC), University of Genova, Genova, Italy

**Keywords:** breast cancer, urinary bladder, metastasis, hematuria, TURB

## Abstract

Breast cancer is one of the most important causes of premature mortality in women worldwide. Around 12% of breast cancer patients will develop metastatic disease, a stage associated with poor prognosis, and only 26% of patients are likely to survive for at least 5 years after being diagnosed. Although the most common sites where breast cancer tends to spread are bones, lungs, brain, and liver, it is important that physicians consider other less frequent organs and viscera, like the bladder, as a target destination. In this article we report our experience with this rare form of metastases and a systematic literature review. We analyzed case reports, case series, and review articles present in PubMED/MEDLINE up to March 2022. We excluded the nonrelevant articles, editorials, letters to the editor, and articles written in other languages. We identified a total of 302 articles, with 200 articles being removed before screening; therefore, the total number of abstracts reviewed was 102. Fifty-five articles were excluded before full text review because they did not meet the inclusion criteria, and one article was not retrievable. Therefore, we included a total of 45 articles in this review. The intention of this review is to highlight the importance of the early detection of bladder metastases and to facilitate the diagnostic process for the responsible physician. The most common signs and symptoms and breast cancer subtype associated with bladder metastases, as well as overall survival after their detection, were all assessed. Bladder metastases from metastatic breast cancer are prevalent in the invasive, lobular breast cancer subtype; most patients present with hematuria (39.5%) and the relative 5-year survival rate is 2%. The main limitations of this review are the low number of cases reported in the literature, clinical and pathological differences between the individual cases, and absence of the control group. This study was not funded.

## Introduction and background

Breast cancer is the most common cancer among women, with the highest incidence in developed countries (1). Actually, it is presumed that one in eight women will develop breast cancer during her lifetime (2). Efficient screening programs and adequate treatments do exist, but unfortunately 20%–30% of women will develop synchronous or metachronous distant metastases, leading to circa 400,000 to 500,000 deaths per year worldwide (3). Furthermore, the fact that 3.5%–10% of all women with breast cancer already have metastatic disease *ab initio* should not be neglected (4). This phenomenon is due to the tumor’s extremely dynamic metastatic potential. As a matter of fact, primary breast cancer is an extremely heterogeneous tumor, can be a reservoir of cancer stem cells, or secretes tumor growth factors such as TGF-beta that favor implantation and growth of metastases (5, 6). This also means that 86% of women, if not metastatic, will be alive 5 years after diagnosis, but at high risk for cancer recurrence (from 13% to 41%, depending on tumor and nodal status of the primary disease) (7). Although considered incurable with poor prognosis, survival rates at 5 years in women with metastatic disease has bettered significantly in the last years, with median overall survival raising from 20 to 26 months (8).

The most important parameters that influence development of metastases include tumor size, histological grade, lymphovascular spread, nodal involvement, presence of hormonal receptors, and human epidermal growth factor receptor-2 (HER2) status. Interestingly, these clinicopathological factors and patterns of breast cancer distant relapse are somehow related. ER- and PR-positive tumors have a tendency to spread to the bone, whereas HER2-positive and triple-negative breast cancers, to the visceral organs, including CNS. Actually, half of breast cancers metastasize to the bone and 25% relapse in the liver and lung. On the contrary, metastases to other distant organs such as the kidneys, spleen, or uterus are uncommon, with the bladder remaining one of the rarest destinations. Literature reports urinary bladder metastases should be taken in consideration when treatment decisions are made, and disease-related complications occur (9). In this article we report our experience with this exceptional event and a review of the literature in order to facilitate the early detection of bladder metastases and management of related complications to the responsible physician.

## Our experience

To date, we have had experience with three metastatic breast cancer patients who presented urinary bladder metastases. Interestingly, all three patients presented painless gross hematuria as the first sign of bladder involvement.

### Case 1

**Figure 1:**
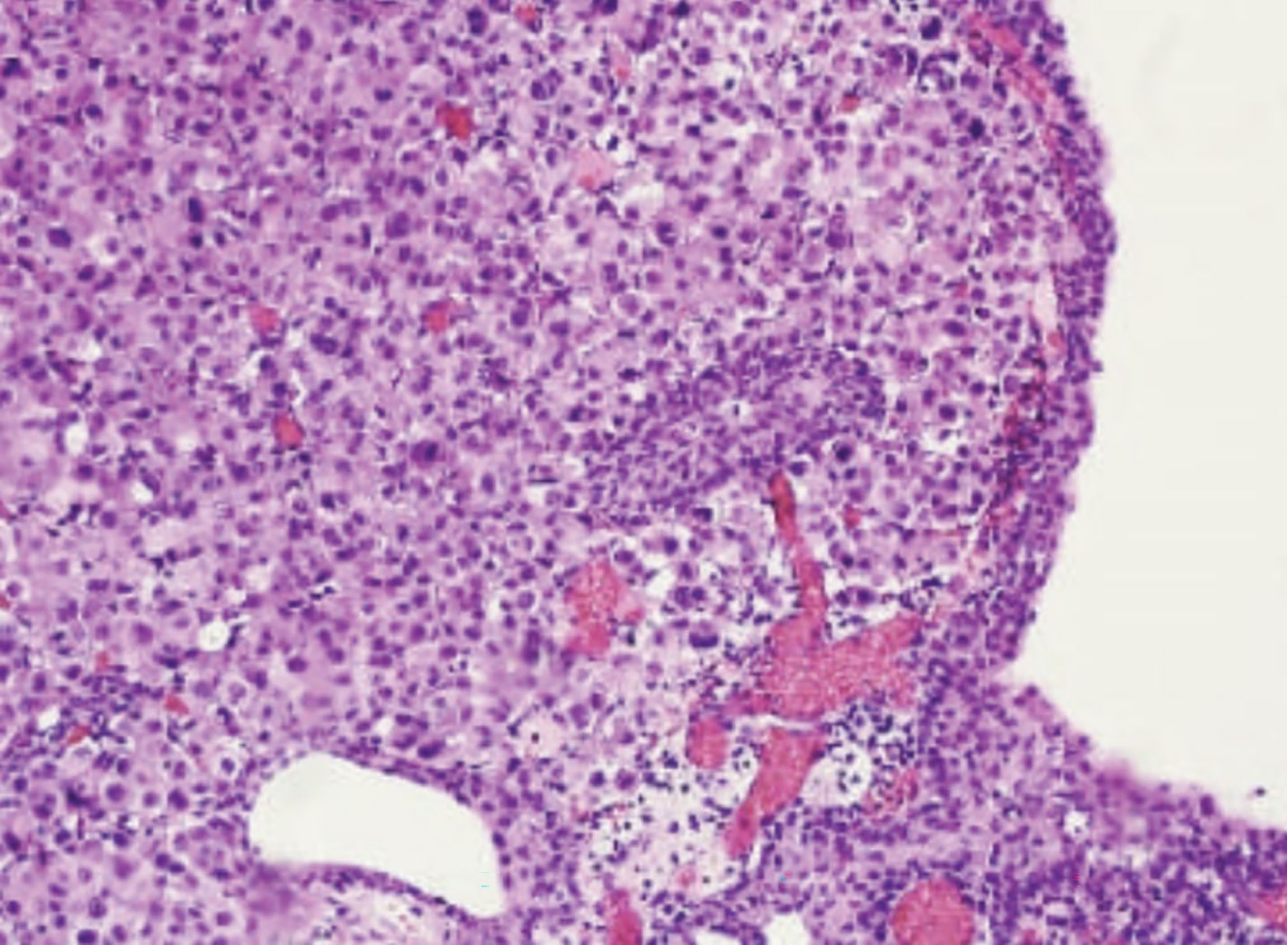
Hematotoxylin-eosin coloration (20x) showing the submucosal layer of the urinary bladder invaded by poorly differentiated, atypical cells with deep infiltration of the muscolaris propria. Urothelial mucosa is completely intact.

**Figure 2:**
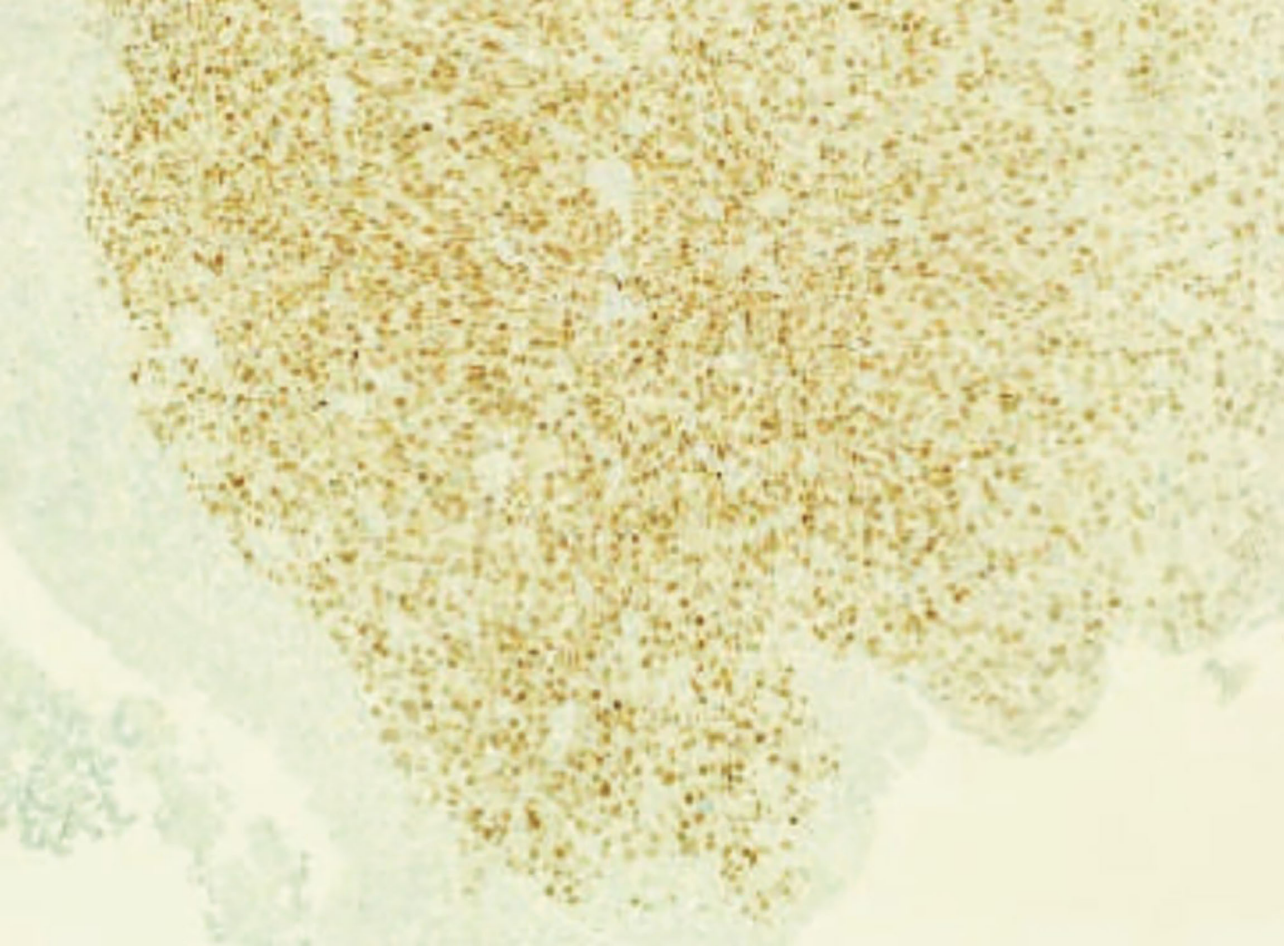
Diffuse positivity for estrogen receptors of muscularis propria on the immunohistochemical examination with bladder mucosa being completely negative.

The first patient that presented this rare form of widespread disease was a 57-year-old Caucasian woman without any family history of breast cancer. Her past medical history revealed multiple episodes of thrombophlebitis in her lower extremities, a profound venous thrombosis due to chronic venous stasis in her right upper extremity, and a myocardial infarction in 2011, treated with angioplasty and coronary stenting. Also, the patient suffered from obesity, hypertension, dyslipidemia, and metabolic syndrome. In 2013, after palpating the nodule in her right breast, a tumor measuring 16 mm was found on the mammogram. She underwent Tru-cut biopsy with a diagnosis of invasive ductal breast carcinoma (95% of tumor cells expressed estrogen receptor (ER), 40% progesterone receptor (PgR), 40% Ki67 and were HER2 negative). Preoperative staging work-up showed an involvement of regional lymph nodes, so she was started on a combined fluorouracil, epirubicin, cyclophosphamide and paclitaxel neoadjuvant chemotherapy. After documented disease regression, mastectomy with ipsilateral axillary lymph nodes dissection was performed. The final pathological examination confirmed luminal B, invasive ductal carcinoma, but also described, despite neoadjuvant chemotherapy, multiple lymph node metastases, with one already having an extranodal extension. Furthermore, final immunohistochemical examination found 95% of tumor cells expressing estrogen receptors (ER), 55% progesterone receptors (PgR), and 1% Ki67, changes possibly due to neoadjuvant treatment (10). Subsequently, she underwent adjuvant radiation therapy associated with oral hormonal therapy, letrozole. Unfortunately, she rapidly progressed, and 36 months after the initial diagnosis, the excisional biopsy of an inguinal lesion confirmed cutaneous breast cancer metastasis. Immunohistochemical coloration showed a persistence of ER (100%) but loss of PgR and increase in Ki-67 activity (40%). Although bone scintigraphy did not show any suspicious lesions, a total-body CT scan evidenced enlarged inguinal lymph nodes, so letrozole was substituted with Fulvestrant. At a 5-year follow-up CT scan, enlarged pelvic lymph nodes, thickened bladder wall and bilateral hydronephrosis were documented. At that point, the patient was enrolled in a phase II clinical trial with CDK4/6 inhibitor Palbociclib as a single agent, or in combination with endocrine therapy, in metastatic, hormone receptor positive (HR+), but HER2 negative, metastatic breast cancer (TREnd trial). During her participation in the trial, she experienced painless, gross hematuria, initially treated as a hemorrhagic cystitis for a couple of months. This was a valid hypothesis considering the previous treatment with cyclophosphamide, and the current therapy with new cytotoxic agents. Nevertheless, after the second episode of gross hematuria, acute urine retention, and serum creatinine increase (1.6 mg/dL, whereas previously she did not suffer from renal insufficiency), she was treated with vesical catheterization and bilateral nephrostomy positioning. She underwent cystoscopy that found suspicious erythematous areas in all bladder regions as well. Shortly afterwards, the patient underwent transurethral bladder resection. The histopathological findings showed muscular invasive anaplastic cells consistent with the primary adenocarcinoma of the breast. Interestingly, the bladder urothelium was completely intact, without any dysplastic or neoplastic changes (**Figures 1**, **2**). Immunohistochemical coloration evidenced positivity for ER, PgR, KRT7, GCDFP 15, and mammoglobin. Moreover, positivity for HER2 appeared and Ki67 immunostaining was markedly increased, indicating the appearance of various mutations and escalation of tumor aggression. Not long after, she started having multiple episodes of malignant pleural effusions for which she was admitted to the palliative care ward. She passed away due to disease-related complications, 5 months after being diagnosed with bladder metastasis.

### Case 2

**Figure 3:**
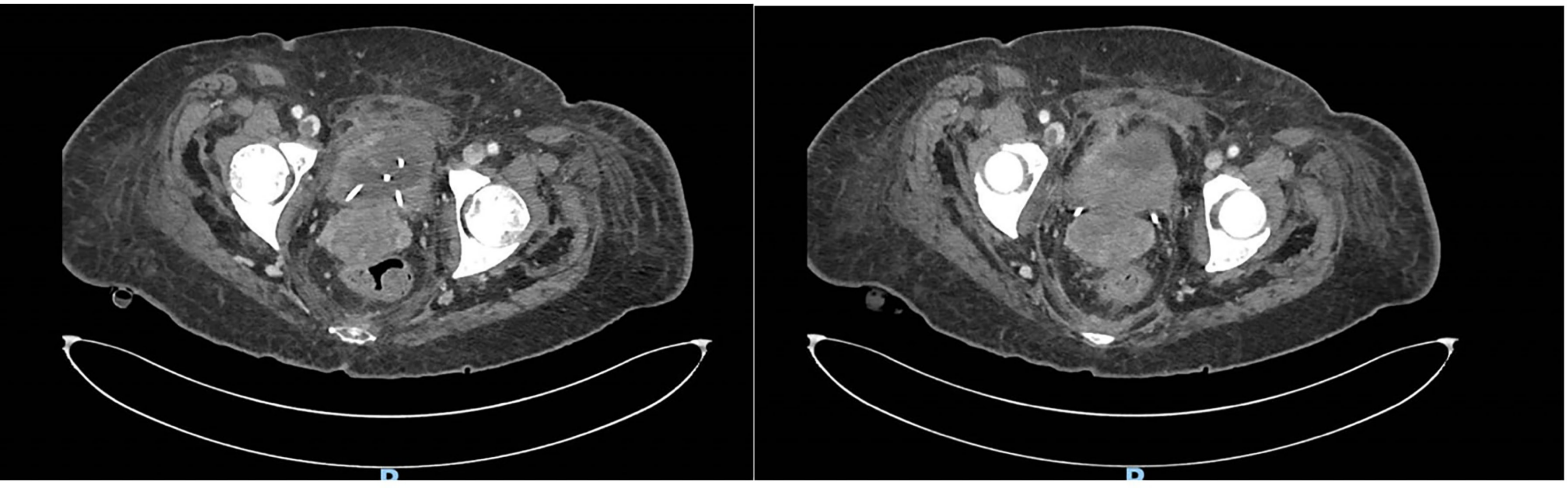
Bladder wall thickening documented on CT scan.

The second patient that came to our attention was a 79-year-old Caucasian woman. Her past medical history did not reveal any significant medical conditions. After finding a lump on her left breast, she was diagnosed with invasive ductal breast cancer on Tru-Cut biopsy (ER, PgR, E-cadherin positive, and HER2 negative) in 2014. She was started on neoadjuvant treatment with fluorouracil, epirubicin, and cyclophosphamide, followed by paclitaxel. After documented disease regression on follow-up imaging, lumpectomy and sentinel node removal was performed. Final pathological examination showed invasive ductal carcinoma with 86% of cancerous cells expressing ER, 78% PgR, 16% Ki-67, but not HER2. Subsequently, she underwent an axillary lymph node dissection with breast cancer metastases found in five of the total 13 excised lymph nodes, and adjuvant radiotherapy of the residual breast tissue. She was put on hormonal therapy with anastrazole for the next 5 years. In 2020, a follow-up, total-body CT scan showed ubiquitous disease dissemination with multiple bone metastases. However, there was no evidence of enlarged lymph nodes or suspicious visceral lesions. Her treatment plan has changed from anatrozole to fulvestrant in combination with abemaciclib, but only for a 6-month period due to inadequate response. In the same year, after being evaluated for DPYD polymorphism that resulted heterozygous for c.2194G>A, she started therapy with cabecitabine, which was also suspended for a lack of benefit. Three months later she received doxorubicin hydrochloride, a third line chemotherapy, followed by a fourth line with Carboplatin associated with Denosumab, which all failed to slow down the course of the disease. The patient developed multiple lymph node localizations, latero-cervical, mediastinic, and pelvic, and a suspicious bladder wall thickening (**Figure 3**). Two months prior to the bladder metastasis diagnosis, she presented with painless gross hematuria, oliguria, and acute renal insufficiency due to lower urinary tract obstruction. This led to hypercreatininemia and hyperkalemia requiring hemodialysis prior to any urological maneuver. Later, after one cycle of hemofiltration, she underwent vesical catheterization and bilateral nephrostomy placement. Transurethral bladder resection was performed with final pathology describing neoplastic infiltration of submucosal and muscular tissue by undifferentiated carcinomatous cells expressing E-cadherin, partial expression of PgR, and a complete loss of ER and KRT5/6. During her hospital stay, left nephrostomy was successfully substituted with ureteral JJ stent, while right ureteral stenting was impossible due to disease infiltration of the intravesical ureter. She passed away 1 month after urinary bladder metastasis diagnosis due to the worsening of her general health status.

### Case 3

Finally, the third patient who presented with urinary bladder metastasis was a 55-year-old Caucasian woman with insignificant past medical history. Her mother was also diagnosed with breast cancer at a young age, but she resulted negative at BRCA genetic testing. She was initially diagnosed in 2015 with invasive, luminal A, lobular breast carcinoma on a Tru-Cut biopsy. She underwent lumpectomy and axillary lymph node dissection immunohistochemistry that revealed cancerous cells expressing 95% of ER, 60% of PgR, and 15% of ki67. Lymph nodes analysis showed a presence of metastases in 15 of the total 16 lymph nodes dissected, multiple already extranodal. She was started on adjuvant chemotherapy with epirubicin, cyclophosphamide, and paclitaxel, followed by radiation therapy of the residual breast tissue. She was given hormone therapy with Letrozole, substituted with Exemestane after a few weeks, due to arthralgia. She progressed 24 months after the initial diagnosis, with the bone being the first metastatic site, and was prescribed with the combination of Fulvestrant and Palpociclib. After only 3 months, her treatment additionally changed to doxorubicin hydrochloride, accompanied by palliative radiation treatment of three bone metastases (sixth left rib, sacrum, and right iliac bone). In 2020, her disease worsened yet again, and was prescribed therapy with Eribulin. In the same year, she was admitted to the ER for painless gross hematuria, anemization and opioid-non-responsive skeletal pain. An urgent CT scan showed very advanced metastatic disease with multiple mediastinic and retroperitoneal metastatic lymph nodes, hepatic metastasis, peritoneal carcinosis, as well as numerous secondary bone lesions. Moreover, a grade I left renal hydronephrosis due to the incarceration of the intravesical ureter and a 15 mm endoluminal bladder lesion were revealed. In order to get the vesical bleeding under control and try and free the ureter from the metastatic disease, she underwent transurethral bladder resection. A final histopathological exam described a breast cancer metastasis with cells organized in small nests and spinnerets expressing weak positivity for PgR, 50% of expression for ER, and 30%–40% for Ki-67. Furthermore, cells started to express HER2 and C-erb-2 oncoproteins. These two oncoproteins were negative on the immunohistochemical examination of the primary tumor, HR was more expressed, and Ki-67 was a lot lower, demonstrating how much aggressive and mutated the secondary lesions were. The patient passed away 2 months after urinary bladder metastasis diagnosis.

**Figure 4:**
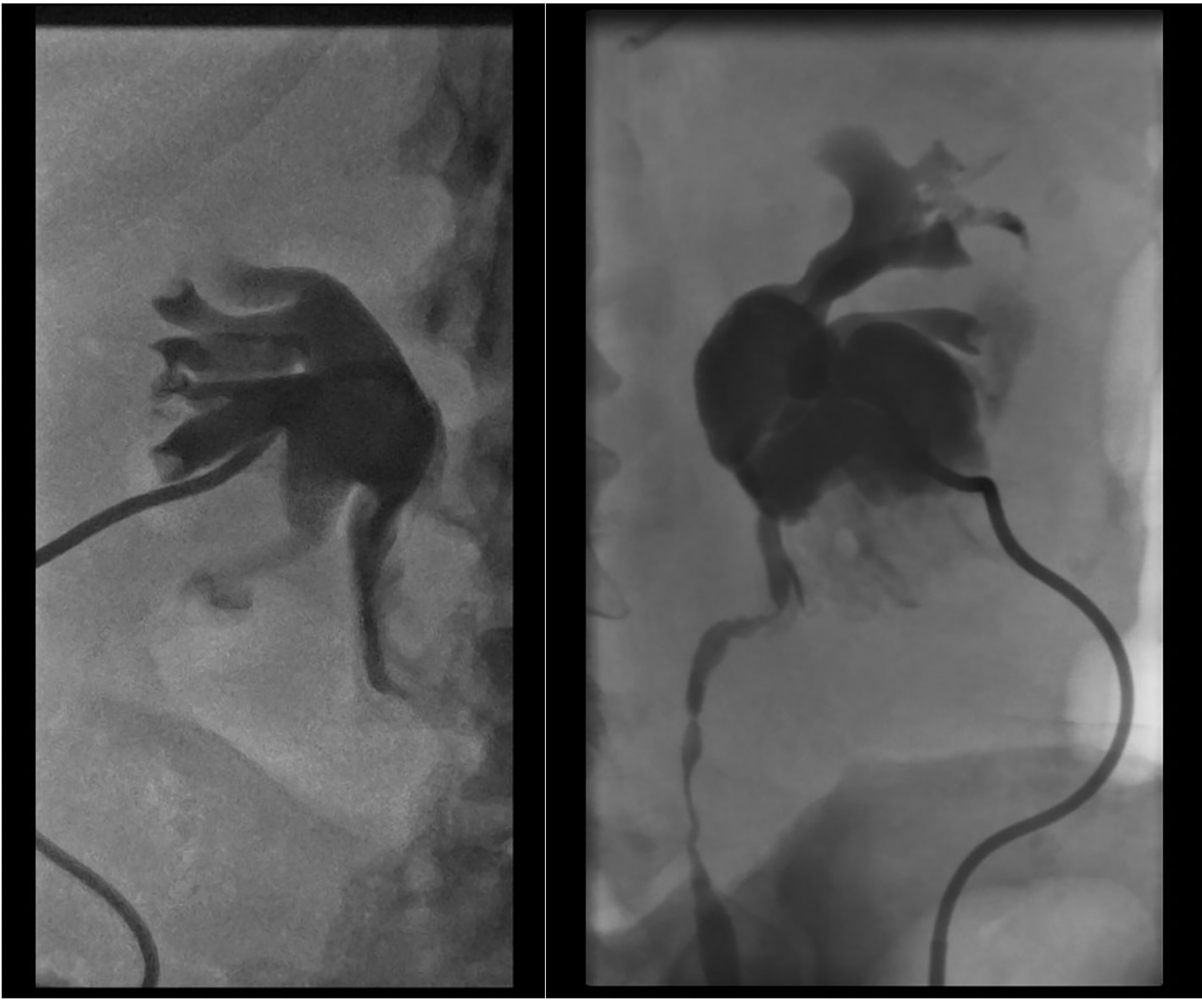
Bilateral hydronephrosis documented on anterograde pyelography during nephrostomy positioning.

Our patients died 66, 84, and 60 months after the initial diagnosis, respectively, and within 5, 1, and 2 months of the bladder metastases resection. Furthermore, all patients were HR-positive and HER2-negative initially, but after multiple local and systemic treatments, secondary lesions that caused rapid disease progression started to show HER2 expression, lower HR, and increased Ki-67 expression with more mitotic activity. Bladder metastases that caused gross hematuria were not evaluated with ultrasound because of its diminished sensitivity in the presence of blood clots, so all three of them underwent CT scan. They underwent transurethral bladder resections to have confirmational pathology and stop the vesical bleeding. Acute kidney injury due to urinary tract obstruction was managed with nephrostomy positioning (**Figure 4**). Unfortunately, all these treatments were purely palliative.

## Materials and methods

We conducted systematic research in the PubMed/MEDLINE database using Medical Subject Headings (MeSH) terms: ((“Breast Neoplasm”[Mesh]) AND (“Neoplasm Metastasis”[Mesh]) AND (“Urinary Bladder”[Mesh])) from 1950 to March 2022, wanting to evaluate epidemiology, histopathology, patterns of spread, signs and symptoms, and treatment of bladder metastases in metastatic breast cancer patients that could help clinicians in their diagnosis and management.

The search was performed by six researchers (R.M., F.B., G.G., A.F., G.T., and F.A.), and any disagreement was resolved after consulting a seventh researcher (G.M.). The initial screening was done based on titles and abstracts.

We included full-text articles, in particular, case reports, case series, and reviews, written in English, French, and Italian languages. After reviewing each manuscript, we also searched its references for any relevant manuscripts missed. We excluded the nonrelevant articles, editorials, letters to the editor, and articles written in other languages.

Data were entered into a Microsoft Excel (Version 2019) database. Descriptive statistics were calculated for all demographic, clinical, and follow-up variables, and reported as median or as a proportion with percentage.

**Figure 5:**
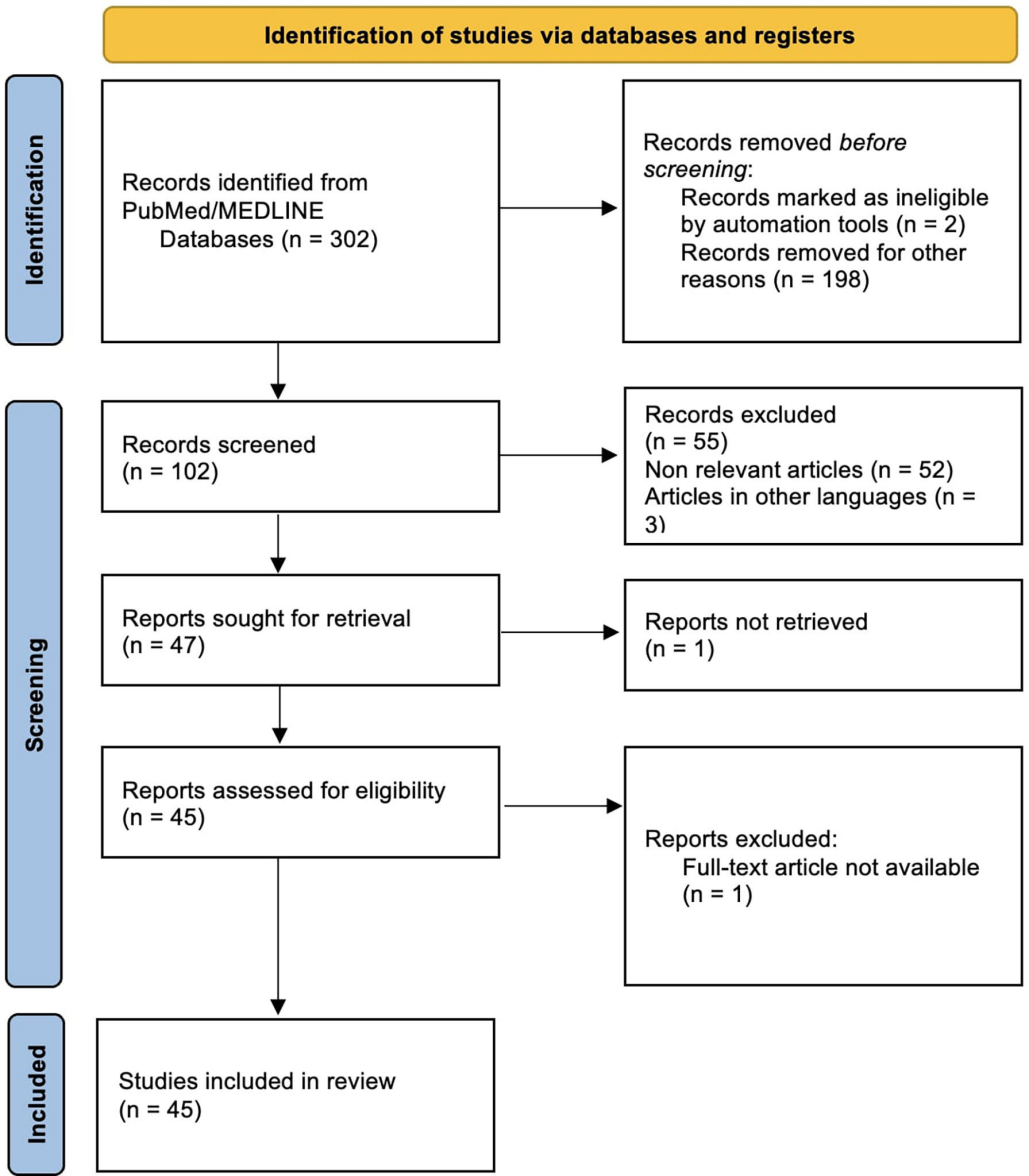
.PRISMA flow chart.

The updated Preferred Reporting Items for Systematic reviews and Meta-Analyses (PRISMA) guidelines (11) were followed for this review, and the flow chart is shown in **Figure 5**.

## Results

By searching the above-mentioned MeSH terms, we identified 302 articles in PubMed/MEDLINE; 200 articles were removed before abstract screening because they were ineligible or not related; therefore, the total number of abstracts reviewed was 102.

During abstract selection, 55 articles were excluded before full text review because they did not meet the inclusion criteria. After abstract selection one full-text article was not retrievable. Therefore, we included a total of 45 articles in this review.

## Discussion

The most common invasive breast cancer histologies are ductal and lobular, representing 50%–75% and 5%–15% of all diagnoses, respectively (12). Breast cancer is further divided into three major subtypes: hormone receptor positive/HER2 negative (HR+/HER2−), HER positive (HER2+), and triple-negative. The main differences between these subtypes are related to prevalence, treatment plan, systemic therapy options and prognoses in stage I and advanced disease. The HR+/HER2- represents 70% of diagnoses and has better prognosis in all stages when compared to HER2+ and triple-negative subtypes. Actually, triple-negative tumors have a high risk for distant recurrence in the first 5 years following diagnosis, and are more frequently diagnosed in younger black and Hispanic patients (13). Metastatic breast cancer remains an incurable disease, and the main goal of therapeutic agents is symptom palliation and prolonging life (13). Regarding patterns of metastatic spread, interestingly, ductal and lobular subtypes have completely different organotropisms when it comes to the uncommon sites. Lobular subtype breast cancer metastases usually appear as nodules, spreading to the parenchymal organs, or as a diffuse, sclerotic-like thickening of serosal surfaces, retroperitoneal space, or walls of the hollow viscera and internal genitalia. Moreover, this ‘diffuse’ pattern of metastatic spread in the retroperitoneum could cause bilateral hydronephrosis and, because of their appearance, remain undetected for a certain period. On the other hand, invasive ductal breast cancer metastases appear almost exclusively as nodules and do not have this tendency to involve the retroperitoneum or viscera (14, 15). Various hypothesis were proposed regarding the pattern of metastatic viscera invasion, but the most accepted one is *via* formation of the tumor emboli. Venous route could possibly use azygos and lumbar veins to reach inferior and superior vena cava, and disseminate distant organs, whilst arterial by bypassing the lungs (16). Another hypothesis is the use of concomitant steroid therapy that causes suppression of the immune system (17).

The urinary bladder is considered a rare target of breast cancer metastases, but secondary lesions to the bladder are actually described in 7% of patients with metastatic breast cancer by some authors (18). A lower number was also reported (4.5%), but in all patients where bladder metastases were clinically not suspected (19). In reality, their actual incidence is still uncertain because in some studies they were not encountered at all (19, 20); also, there are no recent studies. Our literature research found 74 cases described and published from 1950 to date. Finally, elapsed time between initial breast cancer diagnosis and urinary bladder metastases detection varies from within a month up to 30 years (11, 17, 21–62).

Symptoms caused by bladder metastases reported in the literature differ considerably. Most patients do not manifest any signs or symptoms when metastases remain confined to the serosal and muscular layer of the bladder; but when the inner layers, mucosa and submucosa, become infiltrated, common signs and symptoms related to the bladder disease may occur—dysuria, gross or micro hematuria, urge and stress incontinence, with or without flank pain. Hematuria was reported as a first symptom and sign of the disease in 39.5% of cases, urge or stress incontinence and pollakiuria in 32.5%, dysuria associated with flank pain in 16%, whilst 12% of metastatic breast cancer patients were completely asymptomatic, with bladder secondary lesions appearing only on the follow-up imaging. Other, frequent urological diseases that could cause hematuria such as urothelial carcinoma, urolithiasis, and hemorrhagic cystitis caused by drugs, should be excluded first. If all these common conditions were investigated and dismissed, finally, bladder metastases should be suspected. Attentive evaluation of the patient is mandatory, considering that our first patient was treated, once hematuria appeared, for hemorrhagic cystitis, causing a delay in diagnosis and treatment (11, 17, 21–62).

Urogynecological examination should be the first diagnostic approach when evaluating female patients presented with hematuria. A mass could be palpated, particularly if vesical trigone gets involved in the metastatic process, as it happened with our patients, or in the local spread of the primary tumor. On the other hand, hemorrhagic cystitis does not cause any hardening of the bladder walls. Subsequently, if still in doubt, imaging should be ordered. Although all patients with cancer history usually undergo follow-up imaging, and disease progression is frequently caught quite early in asymptomatic patients (26); it becomes more and more obvious that cystoscopy, repeated contrast-enhanced CT urography, and urodynamics should be readily performed when metastatic breast cancer patients present with complicated LUTS and/or hematuria (63). As mentioned previously, on contrast-enhanced CT scan, bladder metastases are generally described as thickening of the bladder wall and could go unobserved for a long time (23); therefore, we should not neglect the importance of cystoscopy. Cystoscopic findings commonly describe a very suspicious erythematous area or, less frequently, a well-defined nodule. At that point, differential diagnosis between primary urothelial tumor and breast cancer metastasis is crucial. Changes in serum tumor-specific biomarkers could help clinicians, as their constant increase could imply breast cancer progression (64).

Immunohistochemical staining is the most important examination for differential diagnosis, especially when the resected tissue contains highly undifferentiated cells. Urothelial carcinoma could express both GATA3 and ER, but not GDFP-15 and mammoglobin, so the standard panel for differential diagnosis includes all of them, associated with PgR (65). Moreover, plasmacytoid urothelial carcinoma could mimic undifferentiated cells of metastatic breast cancer by expressing GATA3, KRT20, and KRT7, but Perrino et al. found that it is always mammoglobin negative (66). Furthermore, biomarker status conversion between primary and metastatic breast cancer tissues is possible, probably due to adjuvant therapy, changing from HR positive to HR negative (46). Biomarker conversion status is an important factor in the management of these patients as it is associated to different therapeutic strategies and shorter survival (67, 68). Although all three of our patients remained weakly positive for the HRs, they lived for 5, 1, and 2 months after the bladder metastases diagnosis, respectively. This could be due to their late recognition, or to an extremely fast disease progression. However, in patients where survival was reported (n = 45), 42% of them were alive 12 months after bladder metastases diagnosis, and only 2% of them 5 years later; reports are attributable to the fact that they are mostly associated with systemic dissemination and multiorgan involvement, although single metastasis to the urinary bladder has been described (46).

The main limitations of this review are the low number of cases reported in the literature, clinical and pathological differences between the individual cases described, and absence of the control group. Nevertheless, we believe that physicians should be quite attentive when managing metastatic breast cancer, particularly the lobular histotype, because bladder metastases could pass unobserved due to their metastatic appearance. Furthermore, they could cause hematuria-related anemization, unpleasant LUTS, abdominal and flank pain, and, more importantly, a decrease in renal function that could prevent patients having their therapy administration. Given the bladder metastases prevalence in these patients, patients too should be informed of this possibility in order to report to their physician any change in micturition as soon as possible, and consequently undergo appropriate imaging and renal function evaluation. The treatment of bladder metastases is usually surgical and only palliative. It generally consists in transurethral bladder resection, ureteral stent positioning or percutaneous drainage of the kidneys, if necessary. The overall survival should be taken in consideration when treating patients in this manner. We should bear in mind the decrease in the quality of life and increase of infection-related risk when, for example, a nephrostomy tube is placed. Generally, bladder metastases are managed quite easily and successfully if there is no delay in their diagnosis, although they are associated with poor prognosis.

This study was not funded.

## Author contributions

RM, GM, and CT contributed to conception and wrote first draft of the review. FB, GG, AF, and GT conducted the research. FA and DP selected the appropriate articles. AR prepared the case reports. RM revised the final version of the manuscript. All authors contributed to manuscript revision, read, and approved the submitted version.

## Conflict of interest

The authors declare that the research was conducted in the absence of any commercial or financial relationships that could be construed as a potential conflict of interest.

## Publisher’s note

All claims expressed in this article are solely those of the authors and do not necessarily represent those of their affiliated organizations, or those of the publisher, the editors and the reviewers. Any product that may be evaluated in this article, or claim that may be made by its manufacturer, is not guaranteed or endorsed by the publisher.

## References

[1] . Available at: http://globocan.iarc.fr/Pages/fact_sheets_cancer.aspx.

[2] Ferrini K Ghelfi F Mannucci R Titta L. Lifestyle, nutrition and breast cancer: facts and presumptions for consideration. Ecancermedicalscience (2015) 9:1–11. doi: 10.3332/ecancer.2015.557 PMC4531134 26284121

[3] Caudle AS Babiera GV. Primary tumor extirpation in stage IV disease: surgical considerations. In: Babiera GV Skoracki RJ Esteva FJ, editors. Advanced therapy of breast disease, 3rd Edition, vol. Vol. 1 . Shelton, CT: PMPH-USA (2012). p. 1001–8.

[4] Khan SA Stewart AK Morrow M. Does aggressive local therapy improve survival in metastatic breast cancer? Surgery (2002) 132:620–6. doi: 10.1067/msy.2002.127544 12407345

[5] Bermas HR Khan SA. Local therapy for the intact breast primary in the presence of metastatic disease. In: Bland KI Copeland EM, editors. The breast, comprehensive management of benign and malignant diseases, 4th Edition, vol. Vol. 2 . Philadelphia: Elsevier Health Sciences (2009). p. 1211–21.

[6] Karnoub AE Dash AB Vo AP Sullivan A Brooks MW Bell GW. Mesenchymal stem cells within tumour stroma promote breast cancer metastasis. Nature (2007) 449(7162):557. doi: 10.1038/nature06188 17914389

[7] Pedersen RN Ozturk Esen B Mellemkjaer L Chrisitansen P Ejlersen B Lash TL. The incidence of breast cancer recurrence 10-32 years after primary diagnosis. JNCI: J Natl Cancer Inst (2022) 114(3):391–9. doi: 10.1093/jnci/djab202 PMC8902439 34747484

[8] Thomas A Khan SA Chrischilles EA Schroeder MC. Initial surgery and survival in stage IV breast cancer in the united states, 1988-2011. JAMA Surg (2016) 151(5):424–31. doi: 10.1001/jamasurg.2015.4539 PMC5844269 26629881

[9] Wei S Siegal GP. Metastatic organotropism: An instrinsic property of breast cancer molecular subtypes. Adv Anatomic Patholo (2017) 24(2):78–81. doi: 10.1097/PAP.0000000000000140 28098572

[10] Choudhary M Ahmed AA Williamson JG. Sole bladder metastasis from breast cancer. J Obstet Gynaecol (2003) 23:212. 12751527

[11] Page MJ McKenzie JE Bossuyt PM Boutron I Hoffmann TC Mulrow CD. The PRISMA 2020 statement: an updated guideline for reporting systematic reviews. BMJ (2021) 372:n71. doi: 10.1136/bmj.n71 33782057 PMC8005924

[12] Dillon D Guidi AJ Schnitt SJ. Pathology of invasive breast cancer. In: Harris JR Lippman ME Morrow M Osborne CK, editors. Diseases of the breast, 5th ed. Philadelphia, PA: Wolters Kluwer Health (2014).

[13] Foulkes WD Smith IE Reis-Filho JS. Triple-negative breast cancer. N Engl J Med (2010) 363(20):1938–48. doi: 10.1056/NEJMra1001389 21067385

[14] Lamovec J Bračkko M. Metastatic pattern of infiltrating lobular carcinoma of the breast: An autopsy study. J Surg Oncol (1991) 48(1):28–33. doi: 10.1002/jso.2930480106 1653879

[15] Borst M Ingold JA. Metastatic pattern of invasive lobular versus invasive ductal carcinoma of the breast. Surgery (1993) 114:637–41. 8211676

[16] Pontes J Edson S Oldford,JR. Metastatic breast carcinoma to the bladder. J Urol (1970) 104(6):839–42. doi: 10.1016/s0022-5347(17)61848-2 5532912

[17] Luczyńska E Pawlik T Chwalibóg A Anioł J Ryś J. Metastatic breast cancer to the bladder case report and review of literature. J Radiol Case Rep (2010) 4:19–26. doi: 10.3941/jrcr.v4i5.395 PMC3303405 22470730

[18] Hagemeister FB Jr Buzdar AU Luna MA Blumenschein GR. Causes of death in breast cancer: a clinicopathological study. Cancer (1980) 46:162–7. doi: 10.1002/1097-0142(19800701)46:1<162::AID-CNCR2820460127>3.0.CO;2-B 7388758

[19] Saphir O Parker ML. Metastasis of primary carcinoma of the breast, with special reference to spleen, adrenal glands and ovaries. Arch Surg (1941) 42:1003–18. doi: 10.1001/archsurg.1941.01210120038004

[20] Warren S Witham EM. Studies on tumour metastasis; distribution of mestastases in cancer of breast. Surg Gynecol Obstet (1933) 57:81–5.

[21] Perez-Mesa C Pickren JW Woodruff MN Mohallatee A. Metastatic carcinoma of the urinary bladder from primary tumors in the mammary gland of female patients. Surg Gynecol Obstet (1965) 121:813–8. 5838289

[22] Haid M Ignatoff J Khandekar JD Graham J Holland J. Urinary bladder metastases from breast carcinoma. Cancer (1980) 46:229–32. doi: 10.1002/1097-0142(19800701)46:1<229::AID-CNCR2820460138>3.0.CO;2-O 7388764

[23] Williams Ma S Moisey CU. Bilateral hydronephrosis secondary to breast carcinoma metastasising to the bladder. Br J Urol (1992) 69:97–8. doi: 10.1111/j.1464-410X.1992.tb15469.x 1737264

[24] Soon PS Lynch W Schwartz P. Breast cancer presenting initially with urinary incontinence: a case of bladder metastasis from breast cancer. Breast (2004) 13:69–71. doi: 10.1016/j.breast.2003.09.005 14759720

[25] Ryan PD Harisinghani M Lerwill MF Kaufman DS. Case 6-2006: a 71-year-old woman with urinary incontinence and a mass in the bladder. N Engl J Med (2006) 354:850–6. doi: 10.1056/NEJMcpc059042 16495398

[26] Ramsey J Beckman EN Winters JC. Breast cancer metastatic to the urinary bladder. Ochsner J (2008) 8:208–12. PMC3096370 21603504

[27] Cormio L Sanguedolce F Di Fino G Massenio P Liuzzi G Ruocco N. Asymptomatic bladder metastasis from breast cancer. Case Rep Urol (2014) 2014:1–3. doi: 10.1155/2014/672591 PMC3971538 24716084

[28] Xiao GQ Chow J Unger PD. Metastatic tumors to the urinary bladder: clinicopathologic study of 11 cases. Int J Surg Pathol (2012) 20:342–8. doi: 10.1177/1066896911428736 22134629

[29] Wang G Zhou C Conklin C Hayes MM Villamil CF Ostry A. Metastatic breast carcinoma to the urinary bladder-a report of 11 cases including a tumor to tumor metastasis. Virchows Arch (2019) 474(3):333–9. doi: 10.1007/s00428-018-02515-3 30607556

[30] Yoneyama K Nakagawa M Hara A. Bladder metastasis from primary breast cancer: a case report. Surg Case Rep (2018) 4(1):73. doi: 10.1186/s40792-018-0484-6 29987656 PMC6037656

[31] Gitau SN Njau A Mwanzi S. Urinary bladder metastasis from breast cancer: a rare cause of hematuria. BJR Case Rep (2020) 6(1):20190048. doi: 10.1259/bjrcr.20190048 32201603 PMC7068102

[32] Jaouani L Zaimi A Al Jarroudi O Brahmi SA Afqir S. Unusual metastasis from breast cancer: Case report. Cureus (2021) 13(10):e18737. doi: 10.7759/cureus.18737 34796050 PMC8589340

[33] Kase AM Menke D Tan W. Breast cancer metastasis to the bladder: a literature review. BMJ Case Rep (2018) 2018:bcr2017222031. doi: 10.1136/bcr-2017-222031 PMC6040494 29954760

[34] Vulcano E Montesano M Battista C Carino R Perrone G Vincenzi B. Urinary complications from breast cancer metastasis: case report and review of the literature. G Chir (2010) 31:243–5. 20615369

[35] Massoud W Ferlicot S Hajj P Awad A Iaaza LA Hammoudi Y. Metastatic breast carcinoma to the bladder. Urol (2009) 74:785–6. doi: 10.1016/j.urology.2009.02.008 19628272

[36] Zagha RM Hamawy KJ. Solitary breast cancer metastasis to the bladder: an unusual occurrence. Urol Oncol (2007) 25:236–9. doi: 10.1016/j.urolonc.2006.05.013 17483021

[37] Elia G Stewart S Makhuli ZN Krenzer BE Mathur S Simon HM. Metastatic breast cancer diagnosed during a work-up for urinary incontinence: A case report. Int Urogynecol J Pelvic Floor Dysfunct (1999) 10:39–42. doi: 10.1007/PL00004013 10207766

[38] Grabstald H Kaufman R. Hydronephrosis secondary to ureteral obstruction by metastatic breast cancer. J Urol (1969) 102:569–76. doi: 10.1016/S0022-5347(17)62202-X 4310510

[39] Ganem EJ Batal JT. Secondary malignant tumors of the urinary bladder metastatic from primary foci in distant organs. J Urol (1956) 75:965–72. doi: 10.1016/S0022-5347(17)66911-8 13320594

[40] Silverstein LI Plaine L Davis JE Kabakow B. Breast carcinoma metastatic to bladder. Urol (1987) 29:544–7. doi: 10.1016/0090-4295(87)90048-3 3554697

[41] Schneidau T Stroumbakis N Choudhury M Eshgi M Mallouh C. Metastatic breast cancer to the bladder. Int Urol Nephrol (1995) 27:297–300. doi: 10.1007/BF02564765 7591593

[42] Poulakis V Witzsch U De Vries R Becht E. Metastatic breast carcinoma to the bladder: 5-year follow up. J Urol (2001) 165:905. doi: 10.1016/S0022-5347(05)66562-7 11176504

[43] Lucas B Simon H Malhaire JP Labat JP. Métastase véscicale d’un cancer du sein. Presse Med (1996) 25:732. 8685141

[44] Feldman PA Madeb R Naroditsky I Halachmi S Nativ O. Metastatic breast cancer to the bladder: a diagnostic challenge and review of the literature. Urol (2002) 59:138. doi: 10.1016/S0090-4295(01)01489-3 11796308

[45] Forster J Agrawal V Anathhanam AJ Spencer N Biyani CS. Breast carcinoma metastasizing to the urinary bladder presenting as bilateral hydronephrosis treated with ureteral stenting and chemotherapy. Urol Oncol (2006) 24:33–5. doi: 10.1016/j.urolonc.2005.07.012 16414490

[46] Lin WC Chen JH. Urinary bladder metastasis from breast cancer with heterogeneic expression of estrogen and progesterone receptors. J Clin Oncol (2007) 25:4308–10. doi: 10.1200/JCO.2007.12.9379 17878482

[47] Nieder C Pawinski A. A case of recurrent breast cancer with solitary metastasis to the urinary bladder. Case Rep Oncol Med (2014) 2014:1–3. doi: 10.1155/2014/931546 PMC3970251 24716053

[48] Al Ibraheemi AA. Case report of metastatic invasive breast lobular carcinoma to the urinary bladder. Int J Hematol Oncol Stem Cell Res (2016) 10:51–5. PMC4818789 27047651

[49] Ghaida RA Ayoub H Nasr R Ghada I Bulbul M. Bladder metastasis from primary breast cancer: a case report and literature review. Cent Eur J Urol (2013) 66:177–84. doi: 10.5173/ceju.2013.02.art17 PMC3936157 24579023

[50] Shah KG Modi PR Rizvi J. Breast carcinoma metastasizing to the urinary bladder and retroperitoneum presenting as acute renal failure. Indian J Urol (2011) 27:135–6. doi: 10.4103/0970-1591.78421 PMC3114575 21716877

[51] Rigatti P Broglia L Montorsi F Maffezzini M Sironi M Radice F. Breast cancer metastases of the urinary bladder. Int J Tissue React (1991) 13:159–63. 1660044

[52] Berger Y Nissenblatt M Salwitz J Lega B. Bladder involvement in metastatic breast carcinoma. J Urol (1992) 147:137–9. doi: 10.1016/S0022-5347(17)37161-6 1729507

[53] Auprich M Augustin H Ruppert A Ploner F Denk H. Bladder metastasis of bilateral breast cancer as first manifestation of metastatic spread: Case report (abstract). BJU Int (2004) 305:10.

[54] Gatti G Zurrida S Gilardi D Bassani G Rosali dos Santos G Luini A. Urinary bladder metastases from breast carcinoma: Review of the literature starting from a clinical case. Tumori (2005) 91:283–6. doi: 10.1177/030089160509100317 16206659

[55] Lawrentschuk N Chan Y Bolton DM. Metastatic breast cancer to the bladder. Breast J (2005) 11:143. doi: 10.1111/j.1075-122X.2005.21427.x 15730462

[56] De Rose AF Balzarini F Mantica G Toncini C Terrone C. Late urinary bladder metastasis from breast cancer. Arch Ital Urol Androl (2019) 91(1):60–2. doi: 10.4081/aiua.2019.1.60 30932435

[57] Khan NAJ Abdallah M Tirona MT. Hormone receptor Positive/HER2 negative breast cancer with isolated bladder metastasis: A rare case. J Investig Med High Impact Case Rep (2021) 9:23247096211022186. doi: 10.1177/23247096211022186 PMC8188964 34096366

[58] Abdel-Razeq R AlMasri R Abunasser M Edaily S Jaber O Khader O. Urinary bladder metastasis from primary breast cancer, a rare and challenging diagnosis. a case report and literature review. Ann Med Surg (Lond) (2022) 76:103455. doi: 10.1016/j.amsu.2022.103455 35308426 PMC8927793

[59] Jordan LA Green L. Late breast cancer metastasis to the urinary bladder presenting with bilateral hydronephrosis. Radiol Case Rep (2018) 13(6):1238–41. doi: 10.1016/j.radcr.2018.08.029 PMC6149194 30258514

[60] Mohammed S Bua AA. Metastatic breast cancer to the urinary bladder in the Caribbean. Case Rep Oncol (2021) 14(3):1586–90. doi: 10.1159/000519971 PMC8647105 34950000

[61] Caputo A Addesso M Fraggetta F D’Antonio A. Hematuria in breast cancer: don’t forget bladder metastases! Pathologica (2022) 114(2):170–3. doi: 10.32074/1591-951X-298 PMC9248250 35481569

[62] Mairy Y Opsomer R Donnez J Van Cangh PJ. Bladder metastasis from breast cancer: 2 cases. Acta Urol Belg (1982) 50:87–90. 7080990

[63] Bettez M Tu LM Carlson K Corcos J Gajewski J Jolivet M. Update: guidelines for adult urinary incontinence collaborative consensus document for the Canadian urological association. Can Urol Assoc J (2012) 6:354–63. doi: 10.5489/cuaj.12248 PMC3478335 23093627

[64] Hamza A Hwang MJ Czerniak BA. Secondary tumors of the bladder: a survival outcome study. Ann Diag Pathol (2020) 48:151593. doi: 10.1016/j.anndiagpath.2020.151593 32836180

[65] Winston T. Metastatic cancer with unknown primary site workup. Medscape (2016). Available at: http://emedicine.medscape.com/article/280505.

[66] Perrino CM Eble J Kao CS. Plasmacytoid/diffuse urothelial carcinoma: a single institution immunohistochemical and molecular study of 69 patients. Hum Pathol (2019) 90:27–36. doi: 10.1016/j.humpath.2019.04.012 31054897

[67] Lower EE Glass EL Bradley DA Blau R Heffelfinger S. Impact of metastatic estrogen receptor and progesterone receptor status on survival. Breast Cancer Res Treat (2005) 90:65–70. doi: 10.1007/s10549-004-2756-z 15770528

[68] Metovic J Abate SO Borella F Vissio E Bertero L Mariscotti G. The lobular neoplasia enigma: management and prognosis in a long follow-up case series. World J Surg Oncol (2021) 19(1):80. doi: 10.1186/s12957-021-02182-w 33736652 PMC7976718

